# The Role of Vision in Maintaining Stroke Synchronization in K2 Crew-Boat Kayaking

**DOI:** 10.3389/fspor.2020.569130

**Published:** 2020-10-08

**Authors:** Pui Wah Kong, Cheryl Sihui Tay, Jing Wen Pan

**Affiliations:** ^1^Physical Education and Sports Science Academic Group, National Institute of Education, Nanyang Technological University, Singapore, Singapore; ^2^Office of Graduate Studies and Professional Learning, National Institute of Education, Nanyang Technological University, Singapore, Singapore

**Keywords:** team boat, paddling, video analysis, offsets, eyes

## Abstract

This study investigated the role of vision in maintaining stroke synchronization in crew-boat sprint kayaking. Sixteen sprint kayakers from a national team were paired into eight two-seater (K2) crews. Each crew paddled at high intensity with the back paddler's eyes open or closed in a randomized order. Using video analysis, stroke synchronization was quantified by the timing offsets between the front and back paddlers at four key positions of the stroke. All crews could paddle continuously without capsize or stopping under both visual conditions. In the absence of vision, neither 200-m performance time (*p* = 0.23, *d* = 0.47, small effect size) nor stroke rate (*p* = 0.41, *d* = 0.31, small effect size) was severely affected. There were no significant effects of vision on stroke synchronization offsets between the front and back paddlers across all key positions (all *p* > 0.05). Highly skilled paddlers likely relied on the kinesthetic perception to maintain the boat synchronization when visual information was not available.

## Introduction

Crew-boats are watercrafts manually propelled by two or more crew members and have historically been used for survival purposes such as transport and war. For example, the traditional Maori war canoe which measures about 36 m in length was manned by 80 paddlers each using a single-bladed paddle (Szanto, 2010). Today, crew-boats are primarily used for leisure or competitive racing in its various forms such as kayaking, rowing, outrigger canoeing, and dragon boat racing.

Crew-boat racing involves a complex interaction of the crew members, the boat, and external environmental factors such as the wind and water conditions (Stambolieva et al., 2012). Although it may appear effortless, paddling in a narrow floating boat while maintaining balance is rather difficult because the beam at the waterline of the boat may be as narrow as 0.31 m (Elliott, 2006). In crew boats, it is challenging to investigate the coordination among the crew members and their interactions with boat and environment (Terrien et al., 2020). A well-synchronized crew would appear to perform strokes at the same rhythm. Stroke synchronization refers to the timing of the different phases in a stroke cycle between members in a crew-boat (King and de Rond, 2011; Tay and Kong, 2018, 2020). Stroke synchronization has been suggested as an important factor for good crew-boat performance in sprint kayaking (Robinson et al., 2011) and rowing (Wing and Woodburn, 1995; Fothergill et al., 2008; King and de Rond, 2011; Cuijpers et al., 2015). Practically, synchronized stroke rhythm is needed due to the close seating proximity between the members. If one member is starting the stroke while another member is ending the stroke, it is highly likely that their paddles or oars would clash. A kayak is propelled by cyclical repetitions of the paddling stroke on alternating sides. This means that the stroke is at some diagonal angle compared to the centreline of the boat, which thus causes yawing motions about the vertical axis. To an observer from far, it would appear that the kayak is moving in a perfectly straight line. Upon closer examination however, the kayak actually moves in a snake-like motion, veering slightly from left to right and vice versa. In the current literature, it remains unclear how stroke synchronization is maintained in a crew-boat.

Wing and Woodburn (1995) proposed two theories on how rhythm transmission occurred in a crew-boat, using an example of an eight-seater rowing boat. The first theory, dubbed the hierarchical dependence structure, suggested a reliance on vision where each crew member tried to mimic the movements of the teammate directly in front. Since an eight-seater rowing boat has rowers stroking on alternate sides (e.g. positions 1, 3, 5, and 7 stroke on the left while positions 2, 4, 6, and 8 stroke on the right), it can be expected that a rower would show higher synchronization with those rowing on the same side as compared with those on the opposite side. However, empirical evidence is inconsistent. For example, one case study which analyzed 19 inter-stroke intervals of a novice crew found stronger correlations between rowers on the same side compared with those on opposite sides (Wing et al., 1992). In contrast, a subsequent study found no difference in stroke synchronization between club level rowers on the same side or on opposite sides (Wing and Woodburn, 1995). The lack of agreement in these case studies questions the relevance of the hierarchical dependence structure theory for rhythm transmission in a crew-boat. An alternative theory was that a common timing source, i.e., the motion of the racing team boat, provided the intrinsic (kinesthetic) feedback needed for movement synchronization (Wing and Woodburn, 1995). This theory is plausible, considering the constraints experienced in a large crew-boat. Due to the length of the boat and the multiple seats involved, not all crew members would be able to visually observe the pacesetter(s) at the front. If each crew member relied solely on observing the teammate in front, there is likely a timing lag which would result in a “caterpillar” effect. Hence, it is possible that there is some reliance on a common timing source, but no supporting data have been published.

In normal circumstances, maintaining balance in a streamlined sprint boat can be achieved by the visual, vestibular, somatosensory, and auditory inputs (Horak et al., 1994; Schaffert et al., 2019). The extent to which how paddlers rely on vision to maintain stroke synchronization in a crew-boat can be better understood by investigating the “eyes closed technique drill,” which is commonly used in crew-boat training. In this drill, all crew members, except for the one responsible for steering, are required to close their eyes and continue with the strokes. Anecdotal evidence suggests that even in the absence of vision, most crews can somewhat coordinate and maintain the stroke synchronization without any major disruptions (e.g., having to stop or capsize). Considering that the crew-boats used in sprint racing have streamlined designs which can be as narrow as 0.37 m, the absence of vision further adds to the difficulty of achieving postural balance and risk of capsizing. Nonetheless, novice athletes with only 1 year of crew-boat experience were observed to perform the drill successfully. Adopting the “eyes closed technique drill” to investigate to role of vision may help clarify how stroke synchronization is maintained in a crew-boat.

Given that it is extremely challenging to examine all members of a large crew boat, the present research study focused on the two-seater K2 kayak which is the smallest unit of a sprint kayaking crew-boat. This study investigated the role of vision in maintaining stroke synchronization in well-trained K2 crew-boat athletes. In a K2, two paddlers are seated in tandem and use double-bladed paddles to perform cyclical repetitions of a forward stroke in phase. In previous studies on well-trained athletes, movement precision can be compromised with augmented external inputs (Schaffert et al., 2020) and that the ability to shift from vision to vestibular and somatosensory inputs was stronger in experienced athletes (Perrin et al., 2002; Paillard et al., 2011). Based on these earlier works, it was hypothesized that when vision of the back paddler of a K2 crew was obscured, stroke synchronization and performance time would worsen.

## Materials and Methods

### Participants

This study complied with the tenets of the Declaration of Helsinki and was approved by the Nanyang Technological University Institutional Review Board (IRB-2014-12-022). All participants gave their written informed consent prior to any data collection. Eight male and eight female sprint kayakers from the Singapore national team participated in this study (Table 1). The inclusion criteria were (1) member of the senior national squad or senior development squad, (2) ranked at least a top six placing in either K1 or K2 class for any sprint distance at the most recent national competition or achieved comparable standards at the national team time trials; and (3) undertaking regular training in the past 3 months prior to the study. The participants were competing internationally and training for 25–30 h per week. They were healthy and injury-free at the time of the study. All crews were either male or female crews, with no mixed sex crews.

**Table 1:** Characteristics of sprint kayakers from the Singapore national team.

	**Male (*n* = 8)**	**Female (*n* = 8)**
Age [year]	23.9 (2.9)	25.6 (3.7)
Height [m]	1.74 (0.05)	1.62 (0.05)
Body mass [kg]	73.8 (3.3)	56.8 (6.8)
Competitive experience [year]	10.4 (1.8)	9.4 (3.3)

*Data are expressed in mean (standard deviation)*.

### Study Design

**Figure 1:**
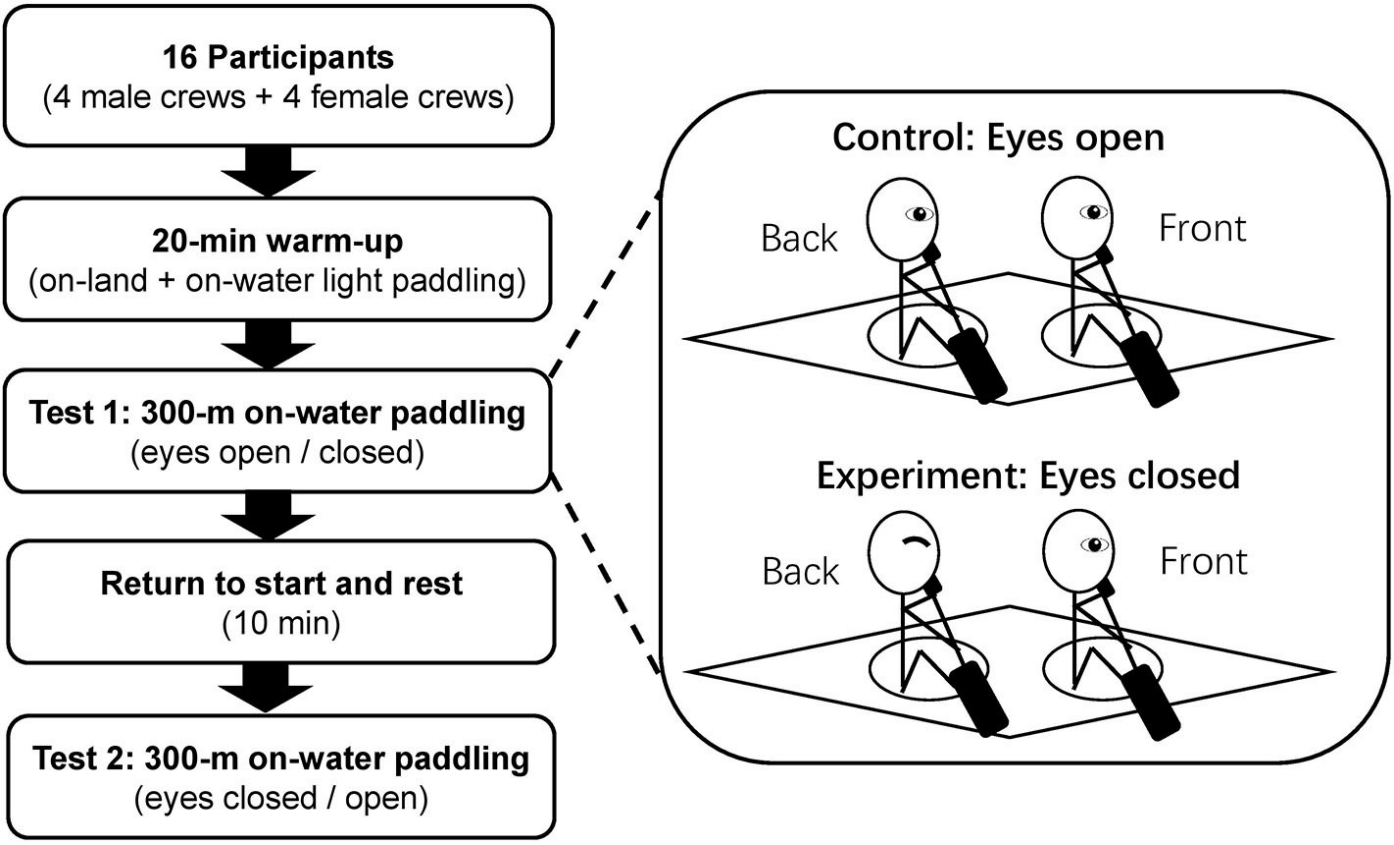
Study flow chart of K2 crews paddled under two visual conditions in a randomized order (control: eyes open and experimental: eyes closed).

This was an experimental study which involved K2 crews paddling at high intensity under two different conditions (eyes open and eyes closed). A randomized and counter-balanced design was applied for the eight crews (16 participants). In the control condition, the eyes of both paddlers were open whereas in the experimental conditions, the paddler at the back seat had his/her eyes closed (Figure 1). Stroke synchronization was measured by timing offsets via video analysis. Within-crew comparisons were conducted to examine the effects of vision on stroke synchronization and performance time.

### Protocol

During a session of crew boat try-outs at the start on the competitive season, the 16 participants were paired into eight single-gender K2 crews. All K2 crews were experienced pairs who regularly trained together in their preferred seat orders. Each crew was assigned a similar model of a sprint kayak K2 weighed to ensure it met the minimum competition weight of 18.0 kg. Should the boat fall short of the minimum weight requirement, additional weights were fixed to the boat (Tay and Kong, 2018). Participants customized the seat and footrest fittings to their preference, used their personal paddles, and wore their regular training attire.

Self-selected warm-ups on land were performed before a 20-min on-water light paddling warmup in their respective K2 crews at a low to moderate intensity. For the actual experiment, each crew paddled two 300-m sets (with eyes open and eyes closed) at specified intensity separated by 10 min of rest. Participants were familiar with the “eyes closed technique drill” during their regular training sessions. To ensure that the back paddler did not cheat by opening his/her eyes, their coach was also on the accompanying speed boat, which was near enough to observe the back paddler carefully. To further avoid the back paddler opening his/her eyes, participants were informed that all trials would be verified with a video camera and any unsatisfactory trial (e.g., opening of eyes) would need to be repeated. The paddling intensity was prescribed at a rating of perceived exertion (RPE) between 15 and 16 on the Borg 6–20 scale, which represents a hard effort (Borg, 1998). The participants in the present study were well-trained athletes in the national team who trained 25–30 h per week and were familiar with the RPE scale as a means of moderating their training intensity. Experimental studies of elite, sub-elite, and international level junior sprint kayakers have also used the Borg 6–20 scale to quantify exercise intensity (van Someren and Oliver, 2002; Sun et al., 2008; Borges et al., 2013). To minimize any environment effects due to wind or water conditions, all trials were performed in the same direction on water.

In the present study, each crew was recorded from the right-hand side sagittal view using a high-speed digital video camera (Casio EX-FH 100, Casio, Tokyo, Japan). The camera was operated at 120 fps from an accompanying speed boat about 5 m away, providing a capture space of ~8.5 m wide (Tay and Kong, 2018, 2020). This sagittal-view recording allowed the researcher to simultaneously observe the positions of the front and back paddlers performing the strokes (Robinson et al., 2011; Tay and Kong, 2018). In general, having distinct left-right asymmetry will cause the boat to “snake” instead of going in a straight line as much as possible, although this could be corrected by additional control of the rudder system with the feet shifting the tiller. Previous experimental work on single-seater touring kayak also reported similar blade forces and impulses between the left and right sides in both competitive and recreational kayakers (Niu et al., 2019). Thus, analyzing the paddling movements on one side was considered an adequate representation of a kayaker.

### Data Processing

To capture the K2 crews at steady state paddling, only the last 200-m section of each 300-m trial was analyzed using the open source freeware Kinovea (Version 0.8.15, available for download at: http://www.kinovea.org). This is because the paddling movements were steadier at the last 200-m section compared with the early 100-m section (Tay and Kong, 2018). First, the performance time was measured based on the tip of the kayak in relation to a pair of adjoining buoy markers demarcating the specific distance. Stroke rate was counted as strokes per min for the back paddler. Next, stroke synchronization within each K2 crew was assessed by the timing offset of the back paddler compared with the front paddler, at four key positions (catch, immersion, extraction, and release) of the stroke based on the paddle blade contact area relative to the water (McDonnell et al., 2012; Tay and Kong, 2018, 2020). “Catch” was defined as the first contact between the paddle blade and water. “Immersion” referred to the instance when the paddle blade was maximally submerged, while “extraction” referred to the last instance where the blade was maximally submerged. Finally, “release” was defined as the last contact between the blade and the water.

The front paddler was taken as the reference from which offsets of the back paddler was calculated. The offset variables were mean offset and percentage offset at each key position of the stroke. A negative offset indicates that the back paddler reaches a position (e.g., catch) before the front paddler. A positive offset indicates that the front paddler reaches a position before the back paddler. The offset is zero if both paddlers reach the same position at the same time. This method of quantifying temporal stroke synchronization does not require camera calibration procedures and has been shown to be highly reliable (Tay and Kong, 2018). All right-side strokes within the 200-m segment were analyzed and the average synchronization offset value was used for subsequent analysis (Tay and Kong, 2020).

### Statistical Analysis

Statistical analyses were performed using SPSS (version 26.0, IBM Corp, Armonk, USA). Normal distribution of the data was confirmed using the Shapiro-Wilk test. A series of paired *t* tests were conducted to compare the differences in performance time and stroke synchronization offsets between the two conditions (eyes open and closed). Statistical significance was set at *p* < 0.05. Results are presented by mean (standard deviation) and 95% confidence intervals. Effect size for the paired *t* test was calculated from the Cohen's d and interpreted as small (0.2 ≤ d < 0.5), medium (0.5 ≤ d < 0.8), or large (d ≥ 0.8).

## Results

All crews could complete the entire segment in their first attempt without any major disruptions such as capsizing or stopping under both eyes open and eyes closed conditions. No repeated trials were needed. All participants verified that they had performed the trials at the instructed intensity of RPE 15–16. On average, 34 right-side strokes (range = 31 to 37, SD = 1.9) were analyzed within the 200-m segment among the crews.

**Figure 2:**
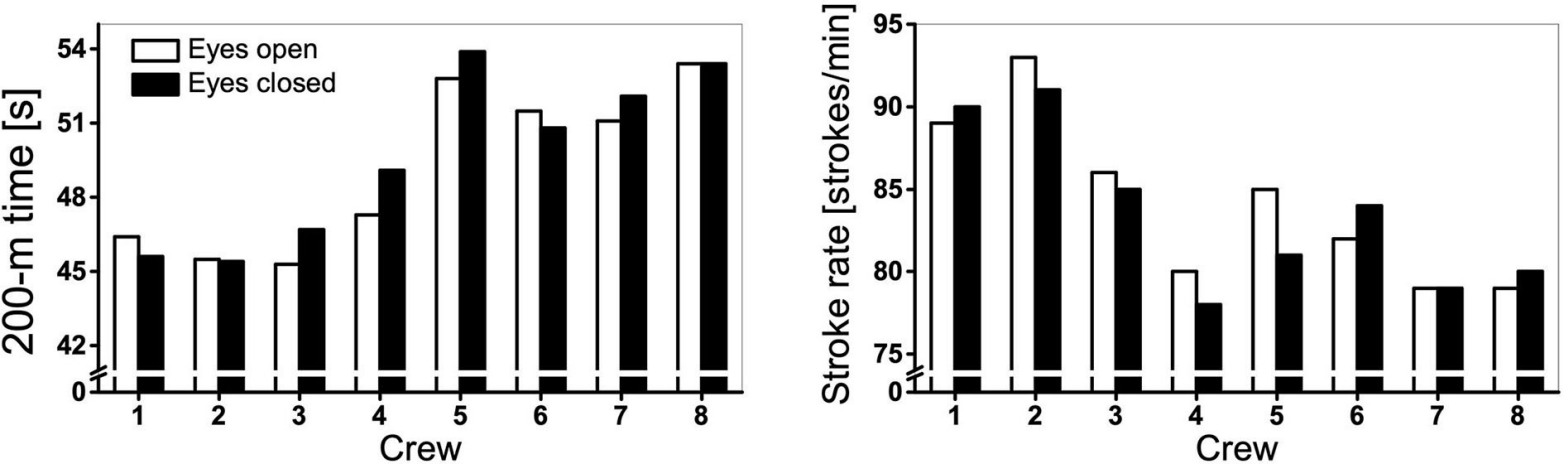
Performance time and stroke rate of eight K2 crews under eyes open and eyes closed conditions of the back paddler.

**Figure 3:**
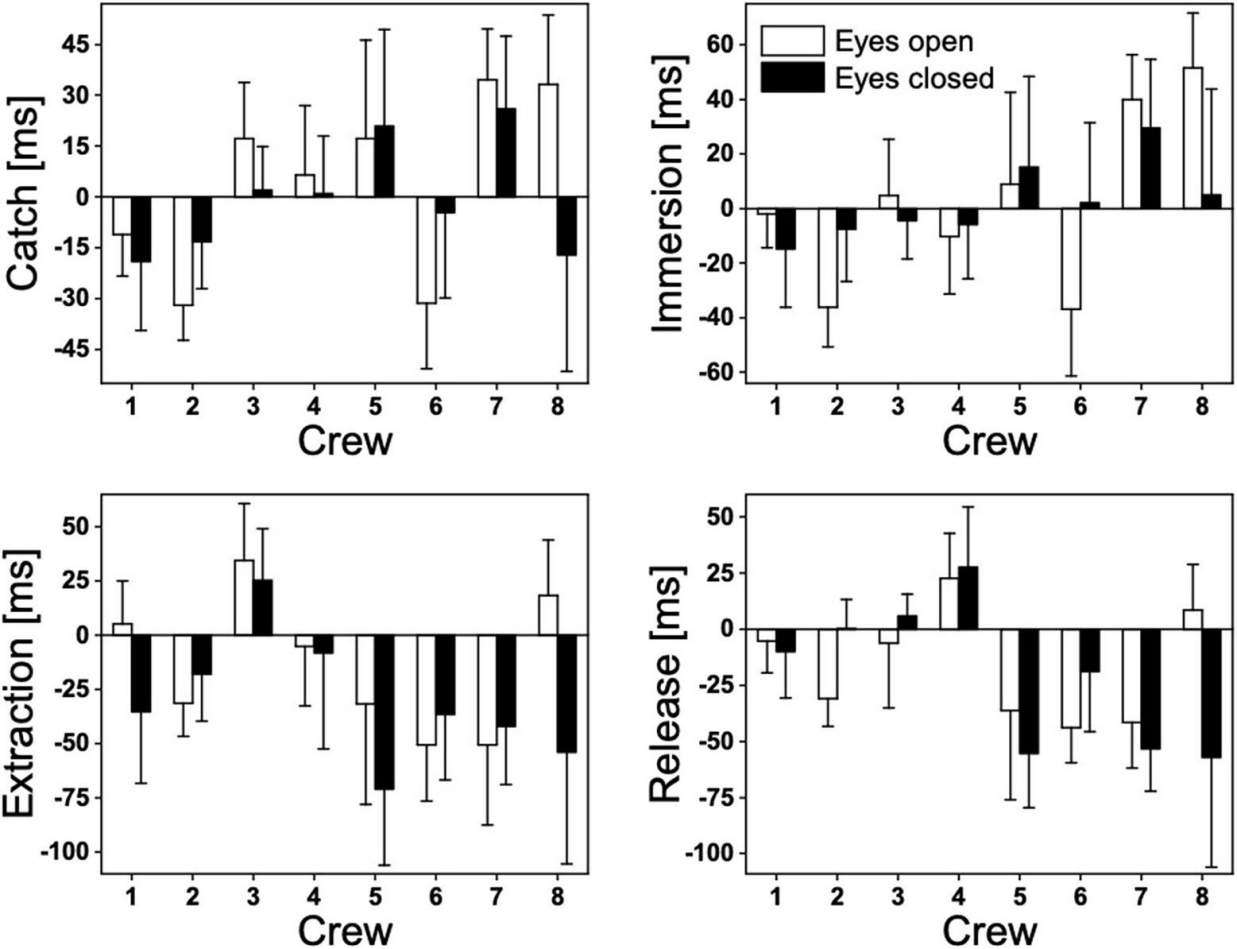
Stroke characteristics of eight K2 crews under eyes open and eyes closed conditions of the back paddler.

There are considerable between-crew variations in the performance time and stroke rate under different visual conditions (Figure 2). As a group, this study did not observe significant differences in the 200-m performance time or stroke rate between conditions (Table 2). With the back paddler's eyes closed, the 200-m performance time (Figure 3) was slower than the eyes open condition in four (crews 3, 4, 5, and 7) out of eight crews. Two crews (crews 2 and 8) exhibited similar performance time regardless of the visual conditions. Interestingly, two crews (crews 1 and 6) were paddling faster under the eyes closed than eyes open conditions. For stroke synchronization offsets, no significant differences were found between the eyes open and eyes closed condition at all four positions. It is notable that the group SD of the offset variables for “catch” and “immersion” were substantially greater under the eyes open than the eyes closed conditions (Table 2).

**Table 2:** Comparison of performance time and stroke characteristics in K2 between eyes open and eyes closed conditions of the back paddler.

	**Eyes open**	**Eyes closed**	**Mean difference (Open – Closed)**	**95% CI**	***p***	**Effect size (d)**
200-m [s]	49.2 (3.4)	49.6 (3.4)	−0.5	[−1.3, 0.4]	0.227	0.468
Stroke rate [strokes/min]	84.1 (5.1)	83.5 (4.9)	0.6	[−1.0, 2.3]	0.405	0.313
Catch offset [ms]	4.3 (26.4)	−0.5 (16.8)	4.8	[−14.6, 24.1]	0.581	0.205
Immersion offset [ms]	2.6 (31.9)	2.1 (14.2)	0.5	[−21.7, 22.7]	0.959	0.019
Extraction offset [ms]	−13.9 (31.6)	−29.9 (29.9)	16.0	[−9.9, 41.9]	0.188	0.516
Release offset [ms]	−16.6 (25.1)	−20.0 (32.0)	3.4	[−21.9, 28.6]	0.761	0.112
Mean offset [ms]	30.1 (10.7)	29.6 (12.4)	0.5	[−9.6, 10.6]	0.910	0.042

*Data are expressed in mean (standard deviation). Stroke rate was counted as strokes per min for the back paddler*.

*CI denotes confidence intervals. Statistical significance was set at p < 0.05. Effect size was calculated from Cohen's d and interpreted as small (0.2 ≤ d < 0.5), medium (0.5 ≤ d < 0.8), or large (d ≥ 0.8)*.

## Discussion

This study investigated the role of vision in maintaining stroke synchronization in K2 crew-boat sprint kayaking during high intensity paddling. The main findings were that performance time and stroke synchronization offsets were not severely altered when vision of the back paddler was obscured. These observations did not support our hypothesis that removing vision would impair K2 crew-boat performance and stroke synchronization.

### The Role of Vision

While maintaining boat balance and coordination can be achieved via visual, vestibular, somatosensory, and auditory inputs (Horak et al., 1994; Schaffert et al., 2019), the hierarchical dependence structure suggested a reliance on vision where the back paddle tried to mimic the movements of the front paddle (Wing and Woodburn, 1995). When vision was removed from the back paddler in the present study, all eight crews could paddle normally without any major disruptions. This interesting finding confirms that well-trained sprint kayak K2 crews did not rely heavily on vision and could perform the “eyes closed technique drill” successfully during high intensity paddling. Surprisingly, the performance times was almost unaffected when the back paddler' eyes were closed (open 49.2 s vs. closed 49.6 s). This indicated that vision did not affect submaximal paddling performance substantially in these well-trained kayakers. In normal circumstances whereby the eyes of both paddlers are open, postural balance can be achieved by the visual, vestibular, and somatosensory inputs (Horak et al., 1994). Findings of this study support that in the absence of one input (e.g., visual), there was a reweighting process of the remaining sensorimotor information to continue and achieve postural balance (Horak et al., 1994). This ability to shift from vision to a combination of the vestibular and somatosensory inputs (proprioception) was previously found to be stronger in experienced athletes (Perrin et al., 2002; Paillard et al., 2011).

When the kayak paddle hits the water, it also produces sound and therefore auditory information may also play a role in the perceptual-motor process (Schaffert et al., 2019). Sounds of actions are found to be informative and movement dynamics, contributing to internal movement presentation (Wolpert et al., 2011). In the present study, the sound produced in aquatic sports may help compensate for the deprivation of the vision in the back paddler. In other aquatic sports such as rowing, a recent study showed that movement precision of elite rowers was comprised with masked hearing (Schaffert et al., 2020). Their findings suggested that natural movement sound in aquatic sports may play a critical role in paddling movement coordination, even at high level of expertise. In the present study, the participants were the kayakers competing for the national team and all of them had roughly 10 years of training experience. When vision was interfered, these well-trained sprint kayakers could reply on vestibular, somatosensory, and auditory inputs to maintain boat balance and paddling rhythm. Currently, while crew-boat kayaking is not included in the Paralympics Games, future investigations could explore whether individuals with visual impairments can participate and benefit from K2 and K4 crew boat kayaking, since good paddling performance can be achieved without relying on the vision of the back paddler.

### Maintaining Stroke Synchronization

The stroke synchronization offsets at all four key positions (catch, immersion, extraction, and release) remained similar between the eyes open and eyes closed conditions in the present study. The consistent synchronization pattern may have contributed to maintaining the performance time despite the vision of back paddler's eyes was removed. An interesting observation is the greater between-subject variabilities of offset timings at “catch” and “immersion” when the eyes were open. This may suggest that vision allows more options or strategies for synchronization during the early stages of a stroke. In the limited literature on K2 crews, one recent study reported that stroke synchronization was largely unaffected when the seat orders of the front and back paddles were switched (Tay and Kong, 2020). The authors concluded that there was no clear effect of seat order on stroke synchronization between the front and back paddlers. Adding to this earlier finding, the present study suggested that vision of the back paddle does not play a strong role in maintaining stroke synchronization in well-trained kayakers.

The lack of vision effect on stroke synchronization is somewhat unexpected, as it contradicts the hierarchical dependence structure theory whereby the back crew member tries to mimic the movements of the teammate directly in front using visual information (Wing et al., 1992). By having the back paddler close his/her eyes, the option of using vision to achieve stroke synchronization was eliminated. It is likely that the paddlers were able to depend more on kinesthetic perception of the cyclical motion of the boat to keep the stroke synchronization. Thus, kinesthetic perception can be viewed as the “common timing source” for the crew members to effectively maintain the boat rhythm (Wing and Woodburn, 1995). While we cannot confirm whether the hierarchical dependence structure theory applies under the eye-open condition, our findings lend support to the common timing source theory in the absence of vision. Possible compensatory mechanisms could come from vestibular inputs, somatosensory inputs, or the natural movement sound when the paddle blade hits the water. Extending from the present work on K2, it would be interesting to see if performance time and stroke synchronization of a four-seater K4 crew can be maintained when the eyes of the second, third, and forth paddlers are all closed. It will also be of interest to explore other situations such as switching seat orders within a crew or mixing members across different crew boats.

### Limitations

One limitation of this study is that the trial intensity was moderated by self-perceived exertion of 15–16, which corresponded to a “hard effort” on the Borg 6–20 scale (Borg, 1982). It is acknowledged that the effects of obscuring vision could have been confounded if the paddlers did not perform both trials at similar effort. Nonetheless, the Borg scale had been used in well-trained sprint kayakers for monitoring training load (Borges et al., 2014), regulating effort in an on-water step test (Place and Billat, 2012), determining training intensities (van Someren and Oliver, 2002), and investigating K2 seat orders (Tay and Kong, 2020). All participants were familiar with using this scale to moderate their paddling intensities as on-water step tests were part of their regular training. After each trial, the participants also verified that they performed the trial at the instructed intensity. To the best of our knowledge, this was the first study which conducted an on-water investigation into the “eye-closed training drill.” As such, while it would have been desirable to test the crews at maximal intensity, there were safety considerations especially for the eye-closed condition. Future studies may consider using heart rate to monitor exercise intensity or instructing crews to paddle at maximal intensity after a period of training.

Secondly, the statistical analysis may be under-power due to the relatively small sample size (16 paddlers, 8 K2 crews). Thus, we reported the effect sizes and confidence intervals to supplement the p-values. Future studies are recommended to include a larger cohort of participants. Lastly, it would also be interested to recruit novice and recreational paddlers who are less well-trained. Having lower proficiency groups may help magnify the differences arising from the role of vision in maintaining stroke synchronization in crew boats.

## Conclusions

Well-trained sprint kayakers were able to paddle continuously at high intensity in a K2 crew boat even when the back paddlers closed the eyes. In the absence of vision, 200-m performance time was not severely affected and there were no significant effects on stroke synchronization. Lending to the common time source theory, paddlers likely relied on the kinesthetic perception to maintain the stroke synchronization when visual information was not available.

## Data Availability Statement

The datasets generated for this study can be found in online repositories. The names of the repository/repositories and accession number(s) can be found in the article/Supplementary Material.

## Ethics Statement

The studies involving human participants were reviewed and approved by Nanyang Technological University Institutional Review Board. Written informed consent to participate in this study was provided by the participants' legal guardian/next of kin.

## Author Contributions

CT and PK originated this project. CT conducted the experiments and processed the data. JP performed statistical analysis. All authors discussed the results and actively contributed to the final manuscript.

## Conflict of Interest

The authors declare that the research was conducted in the absence of any commercial or financial relationships that could be construed as a potential conflict of interest.
